# lncRNA MEG3 restrained the M1 polarization of microglia in acute spinal cord injury through the HuR/A20/NF‐κB axis

**DOI:** 10.1111/bpa.13070

**Published:** 2022-03-25

**Authors:** Heng‐Jun Zhou, Li‐Qing Wang, Ren‐Ya Zhan, Xiu‐Jue Zheng, Jie‐Sheng Zheng

**Affiliations:** ^1^ Department of Neurosurgery The First Affiliated Hospital, College of Medicine, Zhejiang University Hangzhou China; ^2^ Department of Anesthesiology The First Affiliated Hospital, College of Medicine, Zhejiang University Hangzhou China

**Keywords:** acute spinal cord injury, HuR/A20/NF‐κB, lncRNA MEG3, M1 polarization of microglia, neuroinflammation

## Abstract

The M1 polarization of microglia and neuroinflammation restrict the treatment of acute spinal cord injury (ASCI), and long non‐coding ribonucleic acid (lncRNA) maternally expressed gene 3 (MEG3) expression is lessened in ASCI. However, the function and mechanism of lncRNA MEG3 in the M1 polarization of microglia and neuroinflammation in ASCI are unclear. The expressions of lncRNA MEG3 in ASCI mouse spinal cord tissues and lipopolysaccharide (LPS)‐treated primary microglia and BV2 cells were quantified through a quantitative real‐time polymerase chain reaction. In‐vitro assays were conducted to explore the function of lncRNA MEG3 in the M1 polarization of microglia and neuroinflammation in ASCI. RNA degradation, RNA immunoprecipitation, RNA pull‐down, cycloheximide‐chase, and ubiquitination analyses were carried out to probe into the mechanism of lncRNA MEG3 in the M1 polarization of microglia and neuroinflammation in ASCI. The lncRNA MEG3 expression was lessened in the ASCI mouse spinal cord tissues and LPS‐treated primary microglia and BV2 cells, and the overexpression of lncRNA MEG3 restrained the M1 polarization of microglia and the neuroinflammation by regulating the NF‐κB signaling pathway. For the investigation of the potential mechanism of such, the overexpression of lncRNA MEG3 restrained the M1 polarization of microglia through the HuR/A20/NF‐κB axis and boosted the motor function recovery and neuroinflammation relief in the mice with SCI. The overexpression of lncRNA MEG3 restrained the M1 polarization of microglia through the HuR/A20/NF‐κB axis.

## Graphical abstract

A working model was used to reveal the underlying mechanism of lncRNA MEG3 in the M1 polarization of microglia and neuroinflammation.

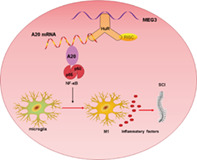

## 1 INTRODUCTION

Spinal cord injury (SCI) usually results in dysfunction of the limbs and causes disability in many cases, mainly including primary and secondary injuries [1, 2]. The secondary injuries involve multiple mechanisms, mainly blood–brain barrier dysfunction, neuroinflammation response, microglia activation, and neuronal death [3, 4], among which the neuroinflammatory response induced by SCI is an important factor that boosts the occurrence and development of SCI [5, 6]. Therefore, the elucidation of the potential mechanism of neuroinflammation is expected to alleviate SCI.

Microglia are the key immune cells in the central nervous system [7], and mainly mediate neuroinflammation after acute spinal cord injury (ASCI), implying that they are the main participants in neuroinflammation [8, 9]. Under normal circumstances, the microglia are usually in a resting state; when SCI occurs, the injured neurons and other cells release a large number of cytokines, such as IL‐1β and TNF‐α, which induce the abnormal activation of microglia and change them from the initial M0 type to M1 or M2 type [10, 11]. M1 microglia usually have a proinflammatory function [12]; as such, restraining the M1 polarization of microglia to reduce the occurrence of neuroinflammation can alleviate ASCI.

Long non‐coding RNAs (lncRNAs) are generally defined as transcripts greater than 200 nucleotides in length that do not have coding capabilities [13] and are bound up with the regulation of a variety of cellular biological processes [14]. Recently, the role of lncRNAs in neurological diseases, including ASCI, has attracted much attention. For instance, Jiang et al. found that lncRNA NHG5 aggravates SCI by increasing the activity of the microglia [15], and Xiang et al. corroborated that knocking down lncRNA Ftx slows down the SCI repair process by boosting the inflammatory response of the microglia [16]. Maternally expressed gene 3 (MEG3) is located in the human chromosome 14q32.3 region [17] and has been recognized as a tumor suppressor gene with low expression in various human diseases [18, 19]. Studies have found that lncRNA MEG3 expression in the serum of traumatic brain injury (TBI) patients is negatively correlated with the level of inflammatory factors, hinting that lncRNA MEG3 can be applied as one of the biomarkers of TBI [20]. Importantly, we analyzed the lncRNA expression profile in mouse SCI dataset GSE45376 and found that the expression of lncRNA MEG3 was lessened on the second and seventh days of SCI, implying that lncRNA MEG3 might be bound up with the occurrence and development of SCI. In the current study, we further corroborated that the overexpression of lncRNA MEG3 restrained the M1 polarization of the microglia and the neuroinflammation induced by lipopolysaccharide (LPS). As such, we firmly believe that lncRNA MEG3 is worth studying.

In the current study, we mainly detected the expression of lncRNA MEG3 in the ASCI mouse spinal cord tissues and LPS‐treated primary microglia and BV2 cells and analyzed the correlation between the expression of lncRNA MEG3 in the serum samples from patients with traumatic SCI and the levels of the TNF‐α, IL‐6, and IL‐1β inflammatory factors. We further probed into the possible mechanism and related signaling pathways of lncRNA MEG3 in regulating the M1 polarization of the microglia in ASCI.

## 2 MATERIALS AND METHODS

### 2.1 Clinical samples

Peripheral blood samples were collected from patients with traumatic SCI (*n* = 36) and healthy controls (*n* = 36) after obtaining such patients' and healthy controls' written informed consent, and the serum samples were centrifuged at 3000 rpm for 10 min. This study was approved by the Ethics Committee of the First Affiliated Hospital, College of Medicine, Zhejiang University.

### 2.2 Construction of a mouse model of acute SCI

Twenty male C57BL/6 mice were obtained from Shanghai Lab, Animal Research Center (Shanghai, China). All the mice were kept in an environment with a 22°C–24°C temperature and 50% humidity and were provided food and water ad libitum. All animal experiment protocols were approved by the Animal Care and Use Committee of the First Affiliated Hospital, College of Medicine, Zhejiang University. The study was carried out in compliance with the ARRIVE guidelines.

The acute SCI mouse model was established based on the method reported in the previous literature [21]. The mice were divided into the sham and SCI groups, and 10 mice were assigned to each group. For the mice in the SCI group, laminectomy was conducted at the T8–T10 thoracic vertebrae without any SCI, and a modified Allen's weight drop apparatus (8 g weight at 50 mm; 8 g × 50 mm) knocking on the exposed spinal cord was applied to induce moderate collision injury. For the mice in the sham group, all the mice underwent laminectomy, and collision injury was not induced.

Moreover, to evaluate the effect of lncRNA MEG3 on mice with SCI, pcDNA‐MEG3 and pcDNA were injected into such mice via intrathecal injection. The mice were divided into the SCI + pcDNA group and the SCI + pcDNA‐MEG3 group, and 10 mice were randomly assigned to each group.

### 2.3 Basso Mouse Scale score

At SCI days 1, 3, 7, 14, 21, and 28, the motor function of the mice was evaluated by Basso Mouse Scale (BMS) score. The scores range from 0 to 9, with 0 representing complete paraplegia and 9 representing a normal function. Each mouse's motor function was analyzed by two independent inspectors who were unaware of the treatment regimen.

### 2.4 Nissl staining

The spinal tissues surrounding the SCI were isolated and fixed with 4% paraformaldehyde. They were then embedded in paraffin wax and sliced into 4‐μm‐thick pieces. The samples were treated with Nissl staining (Beyotime, Wuhan, China) for nearly 5 min, were fixed with neutral balsam (Sigma, San Francisco, CA), and were observed through a microscope (Zeiss, Oberkochen, Germany).

### 2.5 RNA extraction and quantitative real‐time PCR

On the basis of the method reported in the previous literature [22], quantitative real‐time PCR (qRT‐PCR) assay was conducted. Briefly, TRIzol reagent was applied to extract the total RNA from the spinal cord tissues and primary microglia and microglia cell line BV2. PrimeScript RT Reagent Kit (TaKaRa, Dalian, China) was used to synthesize cDNA through the standard procedure indicated by the reagent manufacturer. RT‐PCR was conducted on an ABI 7500 (Applied Biosystems, CA) using SYBR Green RT‐PCR Master Mix (Thermo Fisher Scientific, MA). Glyceraldehyde‐3‐phosphate dehydrogenase was used as an internal reference. The 2^−ΔΔCt^ method was used to measure the relative expressions of lncRNA MEG3, TNF‐α, IL‐6, IL‐1β, A20, and HuR.

### 2.6 Enzyme‐linked immunosorbent assay

The concentrations of TNF‐α, IL‐1β, and IL‐6 in the serum samples were detected by TNF alpha human enzyme‐linked immunosorbent assay (ELISA) Kit (Invitrogen), Human IL‐1β ELISA kit (Immunoway, USA) or Mouse IL‐6 ELISA Kit (Abcam) according to the manufacturer's instructions. Optical density (OD) was measured at 450 nm using a microplate reader (Thermo Scientific).

### 2.7 Isolation and culture of mouse primary microglia

The mouse primary microglia were isolated from the spinal cord tissues using the methods reported in the previous literature [23]. In brief, the mice meninges were carefully isolated and digested with 0.25% trypsin at room temperature for 30 min, and an equal‐volume medium was added to terminate the trypsin digestion. The cells were placed in a single‐cell suspension through repeated pipetting, and were then passed through a sieve (100 μm) and precipitated. Glial cells were resuspended in the medium and were inoculated (1 × 10^5^ cells/ml) in an incubator in 5% CO_2_ at 37°C. The medium was replaced with fresh ingredients once a week, and the mixed glial cell cultures were treated with diluted trypsin to obtain more‐than‐98%‐pure microglial cell cultures.

### 2.8 Detection of CD16/32 and CD206

The membrane proteins CD16/32 (M1) and CD206 (M2) in the primary microglia were detected by flow cytometry assay. The cells were first digested using trypsin, and then washed and re‐suspended with PBS. The cells were incubated with PE‐conjugated monoclonal mouse CD206 antibodies or APC‐conjugated monoclonal mouse CD16/32 antibodies at room temperature in the dark for 30 min. After that, the cells were washed with PBS and re‐suspended. APC and PE‐conjugated monoclonal antibodies with irrelevant specificity were used as negative controls. Each sample were detected by flow cytometry (FACScan, BD Biosciences, USA).

### 2.9 Cell culture

The BV2 cells that were used in this study were purchased from ATCC (USA). They were cultured in Dulbecco's Modified Eagle Medium (DMEM) with the addition of 10% fetal bovine serum (FBS; Gibco, MA) and 100 μg/ml penicillin–streptomycin (Gibco, MA) in 5% CO_2_ at 37°C.

SH‐SY5Y human nerve cells were purchased from ATCC (USA) and were cultured in a 1:1 mixture of DMEM and F12 medium (Gibco, MA) with the addition of 10% FBS (Gibco, MA) in 5% CO_2_ at 37°C.

### 2.10 Cellular localization

Given the previously described method [24], the cytoplasm and nucleus were separated. Following centrifugation at a low temperature, the cytoplasm was obtained from the supernatant after cryogenic centrifugation of the lysates of the primary microglia and BV2 cells. Trizol reagent was applied to extract total cytoplasmic RNA. The precipitate was resuspended in a pyrolysis solution (20 mM HEPES pH 7.9, 80 mM NaCl, 1 mM EDTA and EGTA pH 8.0, 1 mM DTT, 1 mM PMSF) and was centrifuged at 4°C for approximately 10 min at 2800*g*. After the supernatant was discarded, the precipitate was obtained as the nucleus and was then suspended in 1 ml Trizol for RNA isolation. qRT‐PCR was conducted to test the expressions of lncRNA MEG3 in the cytoplasm and nucleus.

### 2.11 Cell transfection and different treatments

To probe into the effects of lncRNA MEG3 on the M1 polarization of microglia and neuroinflammation, the pcDNA‐MEG3 (overexpressing MEG3) or pcDNA (negative control) was transfected into BV2 cells or primary mouse microglia. Specifically, through the standard procedure indicated by the reagent manufacturer, pcDNA‐MEG3 or pcDNA was transfected into BV2 cells or primary mouse microglia using Lipofectamine 2000 (Thermo Fisher Scientific, MA). After 48 h, qRT‐PCR was conducted to verify the transfection efficiency. LPS (1 μg/ml) was also applied to stimulate the cells for approximately 24 h to induce neuroinflammation.

To determine if lncRNA MEG3 affected the M1 polarization of the microglia through the NF‐κB signaling pathway, the BV2 cells or primary mouse microglia transfected with pcDNA‐MEG3 were treated with the NF‐κB signaling pathway activator phorbol myristate acetate (PMA) [25] and then treated with LPS for approximately 24 h. To further probe into whether lncRNA MEG3 regulated the NF‐κB signaling pathway through A20, pcDNA‐MEG3, and/or si‐A20 was transfected into the BV2 cells or primary mouse microglia. The method of cell transfection was the same as the earlier procedure.

To probe into the effect of lncRNA MEG3 on HuR expression, si‐MEG3 was transfected into BV2 cells or primary mouse microglia. To determine if lncRNA MEG3 regulated A20 expression through HuR, pcDNA‐MEG3 and/or pcDNA‐HuR was transfected into BV2 cells or primary mouse microglia. After 48 h, the treatment of the cells with LPS was continued for approximately 24 h.

To determine if lncRNA MEG3 affected the M1 polarization of the microglia through the Hur/A20/NF‐κB pathway, pcDNA‐MEG3 and/or pcDNA‐HuR was transfected into BV2 cells, and the cells were treated with the BAY 11‐7082 NF‐κB pathway inhibitor and were further treated with LPS for approximately 24 h.

### 2.12 Western blot assay

Through the previously described method [26], Western blot assay was conducted. The BV2 cells and mouse spinal cord tissues were gathered, and the total proteins were extracted from them using RIPA lysis buffer (Thermo Fisher Scientific, MA). A total of 25 μg proteins were separated through 10% sodium dodecyl sulfate‐polyacrylamide gel electrophoresis (SDS‐PAGE) and were transferred into the PVDF membranes (Thermo Fisher Scientific, MA). The membranes were then blocked with 5% skim milk and were incubated with the primary antibodies: anti‐ED‐1 (Grbio), anti‐iNOS (ab178945, 1:1000, Abcam), anti‐β‐actin (ab8226, 1 μg/ml, Abcam), anti‐p50 (ab133492, 1:1000, Abcam), anti‐p65 (ab16502, 0.5 μg/ml, Abcam), anti‐p‐p65 (ab183559, 1:1000, Abcam), anti‐A20 (orb159784, 1:1000, Biorbyt), anti‐HuR (ab200342, 1:1000, Abcam), and anti‐IκBα (ab32518, 1:1000, Abcam) overnight at 4°C. The membranes were then incubated with the secondary antibody (ab205718, 1:2000, Abcam) at room temperature for approximately 1 h. ECL Chemiluminescence Kit (Thermo Fisher Scientific, MA) was applied to visualize all the proteins.

### 2.13 Immunofluorescence assay

The spinal cord tissues of the mice and BV2 cells with different treatments were collected. The BV2 cells were fixed with 4% paraformaldehyde and then blocked with 5% bovine serum albumin at room temperature for about 1 h. After that, the cells were incubated overnight with anti‐Iba1 (ab178846, 1:500, Abcam) and anti‐iNOS (ab178945, 1:500, Abcam) at 4°C. The cells were further incubated with the secondary antibodies and were stained with 4′,6‐diamidino‐2‐phenylindole (DAPI) for approximately 15 min. A fluorescent confocal microscope (Olympus, Japan) was used to assess all the images.

In addition, the spinal cord tissues were sliced into 5‐μm‐thick pieces and were further incubated overnight with anti‐Iba1 (ab178846, 1:500, Abcam) and anti‐iNOS (ab178945, 1:500, Abcam) at 4°C. The rest of the experimental operations were conducted in the same way that they were previously conducted.

### 2.14 Transwell co‐culture system

Referring to the previously described methods [27], a transwell co‐culture system was applied to investigate the neuronal death induced by neuroinflammation. In brief, the BV2 cells (5 × 10^4^) were cultured on transwell inserts in a 96‐well plate placed above the SH‐SY5Y neuronal layer.

### 2.15 Cell counting kit‐8

The proliferation ability of the SH‐SY5Y human nerve cells was assessed through cell counting kit‐8 (CCK‐8) assay. Specifically, SH‐SY5Y cells (1 × 10^4^) were added to the 96‐well plates and were cultured overnight, and the medium alone was added as the blank control. Through the standard procedure indicated by the reagent manufacturer, CCK‐8 (Beyotime, Shanghai, China) was used to assess cell proliferation. A 10 μl CCK‐8 solution was added to each well, and the cells were incubated at 37°C for approximately 2 h. A microplate reader (Thermo Fisher Scientific, MA) was used to measure the absorbance values at 450 nm.

### 2.16 Detection of cell apoptosis

Flow cytometry analysis was conducted using the method reported in the previous literature [28]. After the BV2 cells were cultured with the different treatments, the cells were collected for staining. In brief, an Annexin‐V‐FITC Apoptosis Detection Kit (Thermo Fisher Scientific, MA) was used to assess the apoptosis of the BV2 cells. Thereafter, the cells were incubated in the dark with Annexin V/propidium iodide for nearly 30 min, and the percentage of cell apoptosis was tested through flow cytometry (FACScan, BD Biosciences, USA).

### 2.17 RNA immunoprecipitation

RNA Immunoprecipitation (RIP) Kit (Thermo Fisher Scientific, MA) was applied to perform RIP assay. The BV2 cell lysate was incubated with a RIP buffer containing magnetic beads conjugated with the anti‐HuR antibody (Abcam, Cambridge, UK), and normal mouse IgG (Millipore, MA) was used as a negative control. qRT‐PCR was performed to test the relative expression of lncRNA MEG3.

### 2.18 RNA pull‐down

RNA pull‐down assay was conducted to verify the interaction between lncRNA MEG3 and HuR. The details of the experiment were as follows: a large amount of biotin‐labeled MEG3 was synthesized, and the biotinylated MEG3 was captured using streptavidin magnetic beads, through the standard procedure indicated by the reagent manufacturers, and was incubated with BV2 cell lysate. The magnetic beads were washed and the eluted proteins were tested via Western blot.

### 2.19 Cycloheximide‐chase assay

Cycloheximide (CHX) is a commonly used protein synthesis inhibitor [29] and a CHX‐chase assay was conducted using CHX (Sigma, USA). After the si‐MEG3 was transfected into BV2 cells, the cells were mixed with 20 μg/ml CHX and the protein level of HuR was measured via Western blot at 0, 10, 20, and 40 min.

### 2.20 Ubiquitination analysis

After ubiquitin (Thermo Fisher Scientific, MA) and si‐MEG3 were transfected into 293T cells for 36 h, the cells were further treated with 2 mM MG132 for 16 h, followed by protein extraction for Western blot assay. The cell lysates and antibodies were then incubated in immunoprecipitation buffer for 4 h and then incubated with immunomagnetic beads (Millipore, MA) overnight. The eluted protein was tested via Western blot.

### 2.21 Immunohistochemical assay

The spinal cord tissues of mice were harvested and sliced into 5‐μm‐thick pieces, and the slices were deparaffinized and permeabilized. The slices were then incubated with anti‐Ki‐67 antibody (ab16667, 1:200, Abcam) and anti‐p65 (ab16502, 1 μg/ml, Abcam) overnight at 4°C. The slices were further incubated with the secondary antibody (Abcam). After that, the slices were stained with DAPI for approximately 15 min, and the stained slices were imaged using a microscope (Nikon, Japan).

### 2.22 Terminal deoxynucleotidyl transferase dUTP nick end labeling assay

The spinal cord tissues of mice were sliced into 5‐μm‐thick pieces. In accordance with the standard protocol of the reagent manufacturer, a terminal deoxynucleotidyl transferase dUTP nick end labeling (TUNEL) Kit (Roche, Indianapolis, IN) was applied to assess the cell apoptosis in the spinal cord tissues. The spinal cord slices were incubated with a mixture of 50 μl TUNEL at 37°C for approximately 1 h, and the mice spinal cord tissue slices were then stained with DAPI. Microscopy (Leica, Wetzlar, Germany) was conducted for observation and analysis.

### 2.23 RNA fluorescence in situ hybridization

RNA fluorescence in situ hybridization (RNA FISH) was conducted using a RiboTM fluorescent in situ hybridization kit (Ribo Biotechnology Co., Ltd., Guangzhou). After blocking with pre‐hybridization buffer for 30 min, fresh kidney tissue samples were incubated with a hybridization buffer containing 2.5 μM Cy3‐labeled mouse‐Hottip FISH probe (synthesized by Ribo Biotechnology Co., Ltd.) at 37°C overnight. On the second day, the coverslips were counterstained with DAPI for 10 min and photographed under confocal microscopy.

### 2.24 Statistical analysis

SPSS v19.0 was applied to assess all the results of the statistical analyses. At least three independent assays were conducted for each group, and all the values were expressed as mean ± standard deviation. The differences between the two groups were assessed via Student's *t*‐test, and the differences among multiple groups were assessed via one‐way analysis of variance (ANOVA), followed by Tukey's test. Statistical significance was set at *P* < 0.05.

## 3 RESULTS

### 3.1 lncRNA MEG3 expression is decreased in the SCI models

**FIGURE 1:**
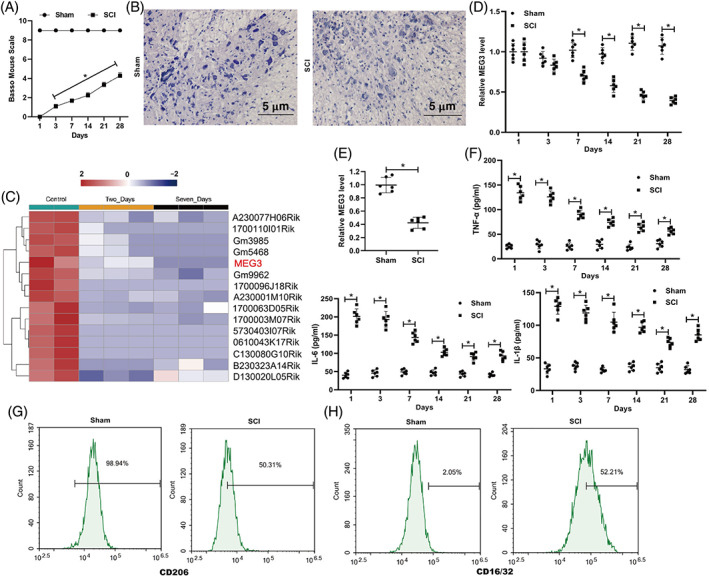
The changes of lncRNA MEG3 expression in the spinal cord injury model in vivo. After the construction of the SCI mouse model using a modified Allen's weight drop apparatus, the spinal cord tissues were isolated. (A) Basso mouse Scale (BMS) score was applied to assess the motor function of mice. (B) Nissl staining was applied to analyze the pathological characteristics of spinal cord tissues in mice (scale bar: 5 μm). (C) GEO database (GSE45376) was applied to the expression profile of lncRNAs. (D) The level of lncRNA MEG3 in serum on days 1, 3, 7, 14, 21, and 28 after SCI has been detected by qRT‐PCR. (E) Quantitative real‐time PCR (qRT‐PCR) was performed to measure the expression of lncRNA MEG3 in the spinal cord tissues. (F) The levels of TNF‐A, IL‐1B, and IL‐6 in serum on days 1, 3, 7, 14, 21, and 28 after SCI has been detected by ELISA. Primary mouse microglia were isolated from the normal mouse and cultured. CD206 (M2) protein expression (G) and CD16/32 (M1) protein expression (H) was measured by flow cytometry assay. *P < 0.05 vs. Sham. SCI, spinal cord injury. Data are represented as the mean ± SD of three independent assays

To determine the potential function of lncRNA MEG3 in SCI, we constructed a mouse model of acute SCI and evaluated the motor function of mice after acquiring SCI using the BMS score. The results indicated that the BMS score became lower in the SCI group (Figure 1A). The results of Nissl staining revealed that the Nissl bodies were reduced after SCI, and some neural shrinkages appeared (Figure 1B). The GEO database (GSE45376) showed that compared with the control group, the lncRNA MEG3 expression was decreased on SCI days 2 and 7 (Figure 1C). qRT‐PCR showed that lncRNA MEG3 level in serum was decreased gradually from the seventh day with the extension of injury time (Figure 1D). As shown in Figure 1E, the lncRNA MEG3 expression in the spinal cord tissues was also confirmed to have decreased in the SCI group, hinting that lncRNA MEG3 might be bound up with the development of SCI. In addition, the expressions of TNF‐α, IL‐6, and IL‐1β in the SCI group were higher than that in the Sham group, and decreased gradually from the first day with the extension of injury time (Figure 1F). The protein expression of CD206 in the primary mouse microglia was markedly downregulated in the SCI group, while CD16/32 protein expression was upregulated (Figure 1G,H).

**FIGURE 2:**
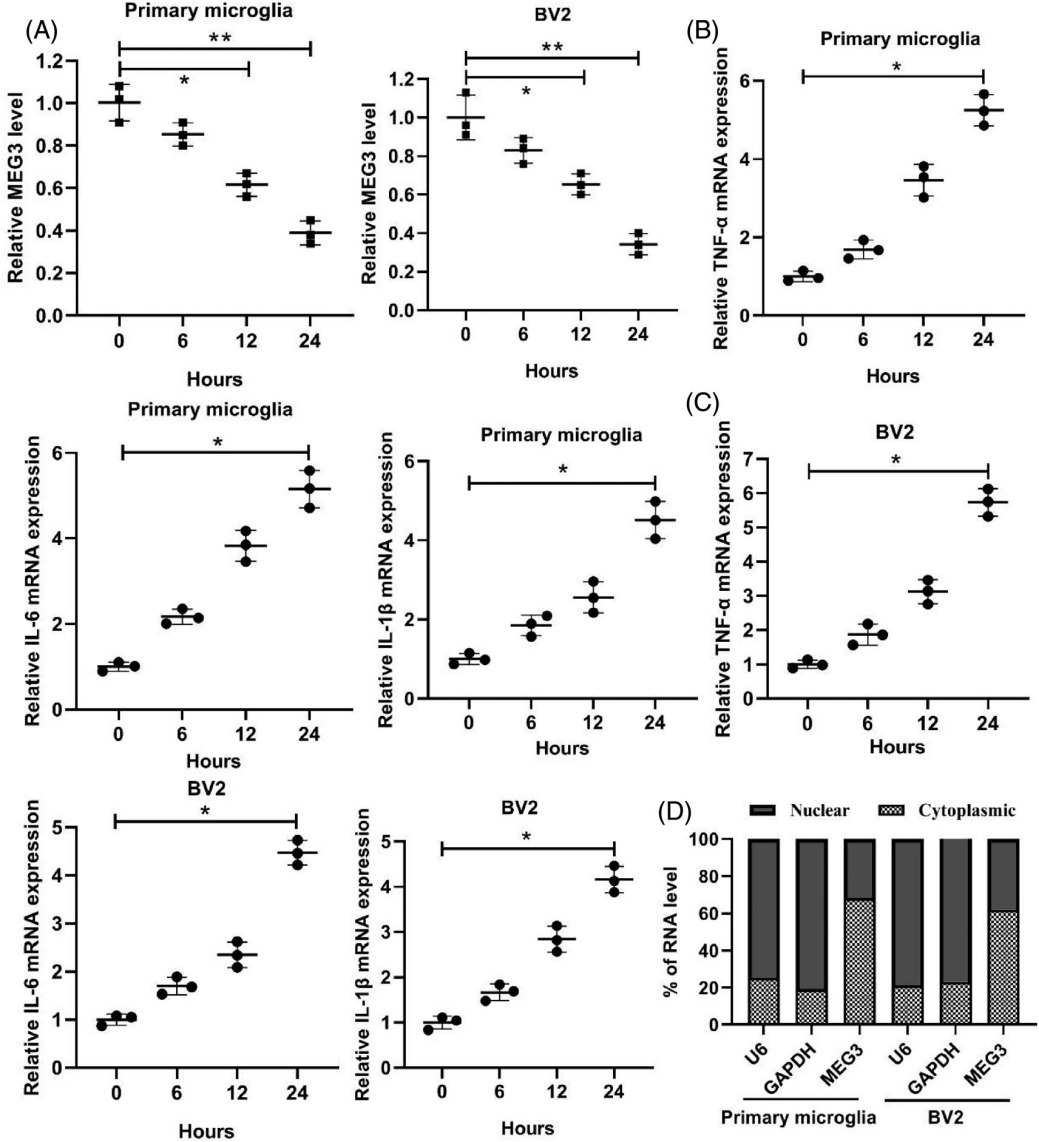
The changes of lncRNA MEG3 expression in the spinal cord injury model in vitro. (A) Primary mouse microglia were isolated from the normal mouse and cultured, and mouse microglia cell line BV2 was also cultured, and then treated the cells with 1 μg/ml LPS for 0, 6, 12, and 24 h. qRT‐PCR was applied to test the expression of lncRNA MEG3. (B) qRT‐PCR was applied to detect the expressions of TNF‐α, IL‐6, and IL‐1β in the primary microglia. (C) qRT‐PCR was applied to detect the expressions of TNF‐α, IL‐6, and IL‐1β in the BV2 cells. (D) The distribution of lncRNA MEG3 in mouse primary microglia and BV2 cells by qRT‐PCR. *P < 0.05, **P < 0.01 vs. Control (0 h). Data are represented as the mean ± SD of three independent assays

As in the in vitro experiments, after the mouse primary microglia and mouse microglia cell line BV2 were treated with LPS for different times, the qRT‐PCR analysis revealed that the lncRNA MEG3 expression was gradually lessened with the extension of the LPS treatment time (Figure 2A). Moreover, the lncRNA MEG3 expression was lessened in the serum samples from the patients with traumatic SCI (Figure S1A). Furthermore, the expressions of TNF‐α, IL‐6, and IL‐1β gradually increased with the extension of the LPS treatment time (Figure 2B,C), and the TNF‐α, IL‐6, and IL‐1β concentrations increased in the serum samples from the patients with traumatic SCI (Figure S1B). The expression of lncRNA MEG3 in the serum samples from the traumatic SCI patients was also negatively correlated with the TNF‐α, IL‐6, and IL‐1β levels (Figure S1C). In addition, we analyzed the distribution of lncRNA MEG3 in mouse primary microglia and BV2 cells and found that lncRNA MEG3 was mainly distributed in the cytoplasm (Figure 2D). These data corroborated that the expression of lncRNA MEG3 was lessened in SCI.

### 3.2 lncRNA MEG3 restrains the M1 polarization of microglia and neuroinflammation induced by LPS

**FIGURE 3:**
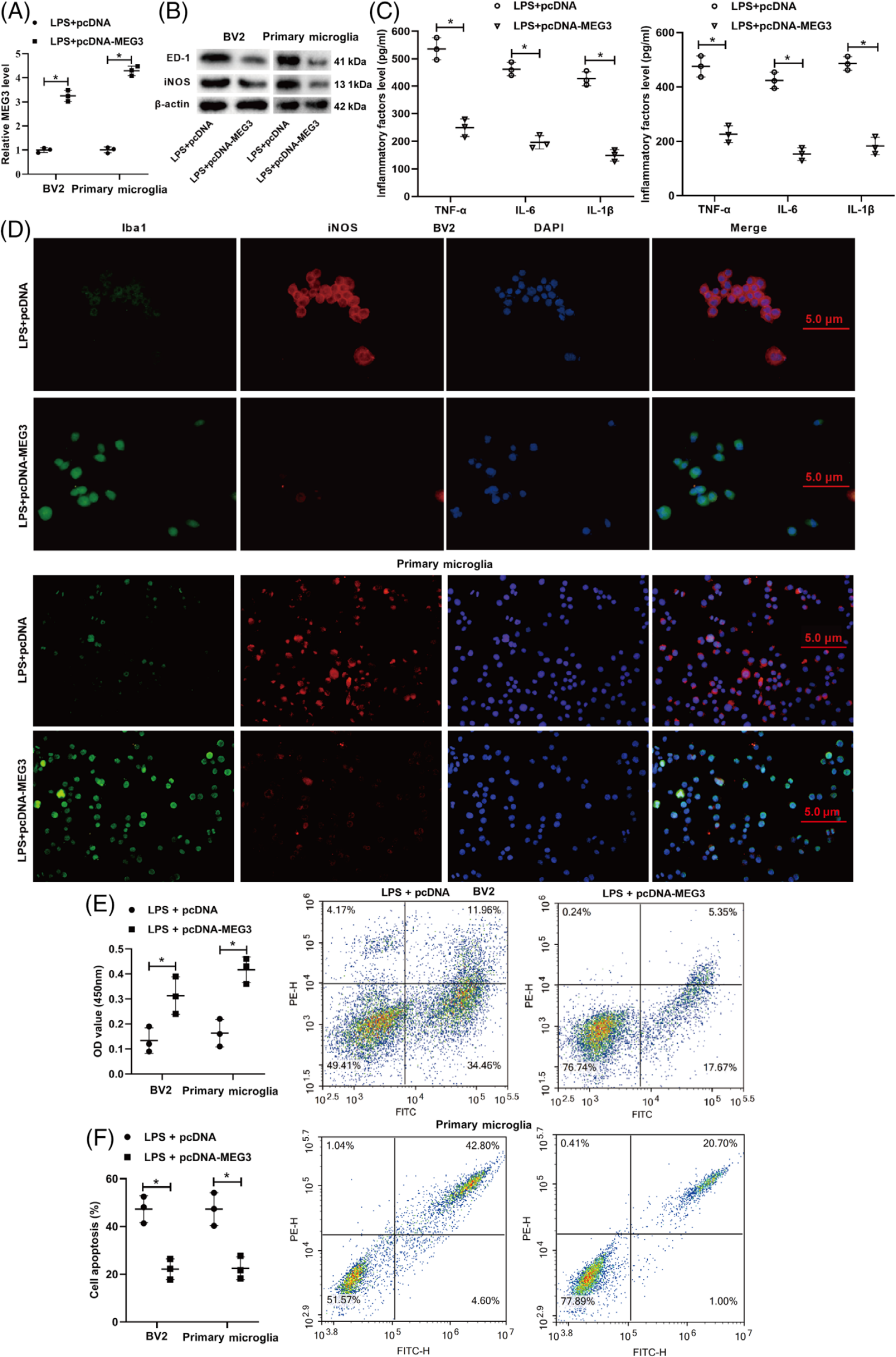
Influence of lncRNA MEG3 in the M1 polarization and neuroinflammation of microglia caused by LPS. (A) pcDNA, pcDNA‐MEG3 was transfected into BV2 cells and spinal primary microglia for 48 h, and then treated the cells with LPS for 24 h. qRT‐PCR was applied to test the expression of lncRNA MEG3. (B) Western blot was applied to measure the protein levels of ED‐1 (a marker of microglial activation) and iNOS (M1 polarization marker of microglia). (C) Enzyme‐linked immunosorbent assay (ELISA) was applied to test the concentrations of inflammatory molecules TNF‐α, IL‐6, and IL‐1β. (D) Immunofluorescence assay was applied to analyze the expression of Iba‐1 (a marker for microglia) (scale bar: 5 μm). BV2 cells and spinal primary microglia transfected with pcDNA‐MEG3 were treated with LPS, and the BV2 cells and spinal primary microglia were co‐incubated with human nerve cells SH‐SY5Y by the Transwell co‐culture system for 12 h. The schematic diagram of co‐culture. (E) Cell counting kit‐8 (CCK‐8) was applied to assess the proliferation ability of SH‐SY5Y cells. (F) Flow cytometry was applied to measure the apoptosis ability of SH‐SY5Y cells. *P < 0.05 vs. LPS + pcDNA. LPS, lipopolysaccharide. Data are represented as the mean ± SD of three independent assays

To investigate the effect of lncRNA MEG3 on LPS‐induced microglial polarization and neuroinflammation, pcDNA‐MEG3 was transfected into BV2 cells and spinal primary microglia, and then the cells were treated with LPS. As shown in Figure 3A, lncRNA MEG3 was successfully overexpressed in the BV2 cells and spinal primary microglia. ED‐1 is one of the markers of microglia activation [30], and iNOS is the M1 polarization marker of microglia [31]. The Western blot assay indicated that the protein levels of ED‐1 and iNOS became lower after the transfection with pcDNA‐MEG3 (Figure 3B). In addition, the enzyme‐linked immunoassay results indicated that the concentrations of the TNF‐α, IL‐6, and IL‐1β inflammatory molecules were reduced in the cell supernatant (Figure 3C).

Iba‐1 is a routine marker for microglia [32]. The immunofluorescence further clarified that the Iba‐1 expression increased and the iNOS expression decreased after the transfection with pcDNA‐MEG3, implying that lncRNA MEG3 restrained the M1 polarization of microglia induced by LPS (Figure 3D). The BV2 cells and spinal primary microglia were transfected with pcDNA‐MEG3 were treated with LPS and were co‐incubated with the SH‐SY5Y human nerve cells through the Transwell co‐culture system for 12 h. The CCK‐8 assay indicated that the proliferation ability of SH‐SY5Y was boosted after the transfection with pcDNA‐MEG3 (Figure 3E) whereas the flow cytometry results showed that the apoptosis ability of SH‐SY5Y was restrained after the transfection with pcDNA‐MEG3 (Figure 3F). In general, lncRNA MEG3 inhibited the M1 polarization of microglia and the neuroinflammation caused by LPS.

### 3.3 lncRNA MEG3 affects the M1 polarization of microglia by regulating the NF‐κB signaling pathway

**FIGURE 4:**
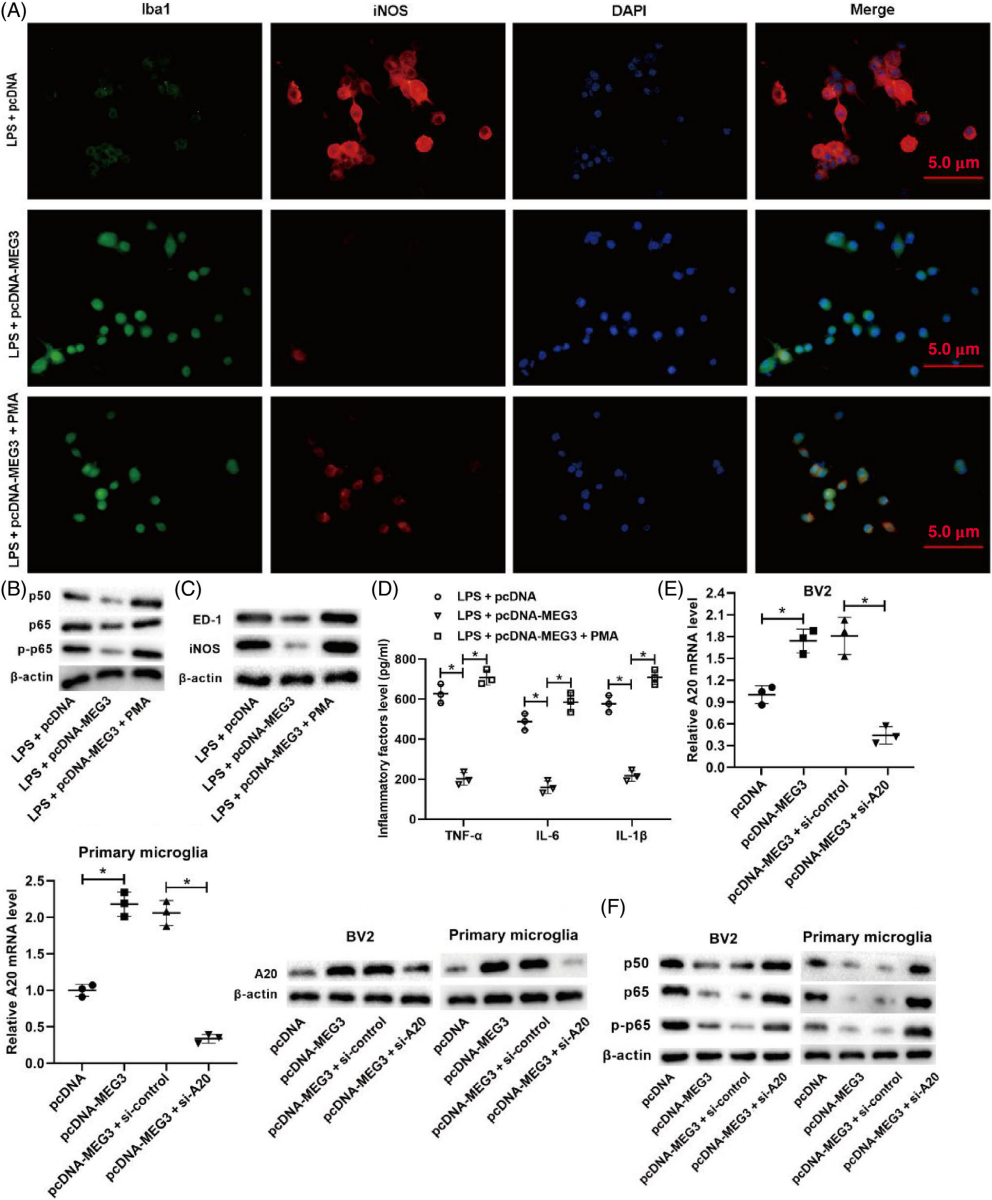
Effect of lncRNA MEG3 on the M1 polarization of microglia by influencing the NF‐κB signaling pathway. BV2 cells transfected with pcDNA‐MEG3 were treated with phorbol myristate acetate (PMA, NF‐κB signaling pathway activator) and then treated the cells with LPS for 24 h. (A) Immunofluorescence analysis was conducted to detect the expressions of ED‐1 and iNOS (scale bar: 5 μm). (B) Western blot was performed to quantify the protein levels of NF‐κB signaling pathway‐related molecules p50, p65, and p‐p65. (C) Western blot was performed to measure the protein levels of ED‐1 and iNOS. (D) ELISA was performed to measure the concentrations of TNF‐α, IL‐6, and IL‐1β. (E) pcDNA‐MEG3 and/or si‐A20 was transfected into BV2 cells and primary microglia. qRT‐PCR and Western blot were applied to test the mRNA and protein levels of A20 (an inhibitor of the NF‐κB signaling pathway). (F) Western blot was conducted to measure the protein levels of p50, p65, and p‐p65. *P < 0.05 vs. LPS + pcDNA, LPS + pcDNA‐MEG3, pcDNA, or pcDNA‐MEG3 + si‐control. PMA, phorbol myristate acetate. Data are represented as the mean ± SD of three independent assays

A previous study has shown that the nuclear transcription factor NF‐κB plays a key role in the process of M1 polarization of the microglia to cytokine release [33]. On the basis of this and the conclusions shown in Figure 3, we speculated whether lncRNA MEG3 is bound up with the regulation of the NF‐κB signaling pathway. Thus, we treated the BV2 cells transfected with pcDNA‐MEG3 with PMA (NF‐κB signaling pathway activator) and then treated the cells with LPS for 24 h. As shown in Figure 4A, the immunofluorescence analysis corroborated that the overexpression of lncRNA MEG3 increased the Iba‐1 expression and decreased the iNOS expression, but these trends were reversed after the treatment with PMA. The overexpression of lncRNA MEG3 lowered the protein levels of the NF‐κB‐signaling‐pathway‐related molecules p50, p65, and p‐p65, but this trend was reversed after the treatment with PMA (Figure 4B). In addition, the overexpression of lncRNA MEG3 lowered the protein levels of ED‐1 and iNOS, but this trend was reversed after the treatment with PMA (Figure 4C). Moreover, the overexpression of lncRNA MEG3 decreased the TNF‐α, IL‐6, and IL‐1β concentrations, but this trend was reversed after the treatment with PMA (Figure 4D).

A20 is a common inhibitor of the NF‐κB signaling pathway [34], which plays an inflammatory role by repressing NF‐κB activation and entry into the nucleus. Based on the aforementioned findings, we continued to probe into whether lncRNA MEG3 could regulate the NF‐κB signaling pathway through A20. For this, pcDNA‐MEG3 and/or si‐A20 was transfected into BV2 cells and primary microglia. As shown in Figure 4E, the overexpression of lncRNA MEG3 raised the messenger RNA (mRNA) and protein levels of A20, but this trend was reversed after the interference with A20. Furthermore, the overexpression of lncRNA MEG3 lowered the protein levels of p50, p65, and p‐p65, but this trend was reversed after the interference with A20 (Figure 4F). Overall, these data showed that lncRNA MEG3 restrained the M1 polarization of microglia through the inactivation of the NF‐κB signaling pathway.

### 3.4 HuR boosts the degradation of A20 mRNA and the verification of the interaction between lncRNA MEG3 and HuR protein

**FIGURE 5:**
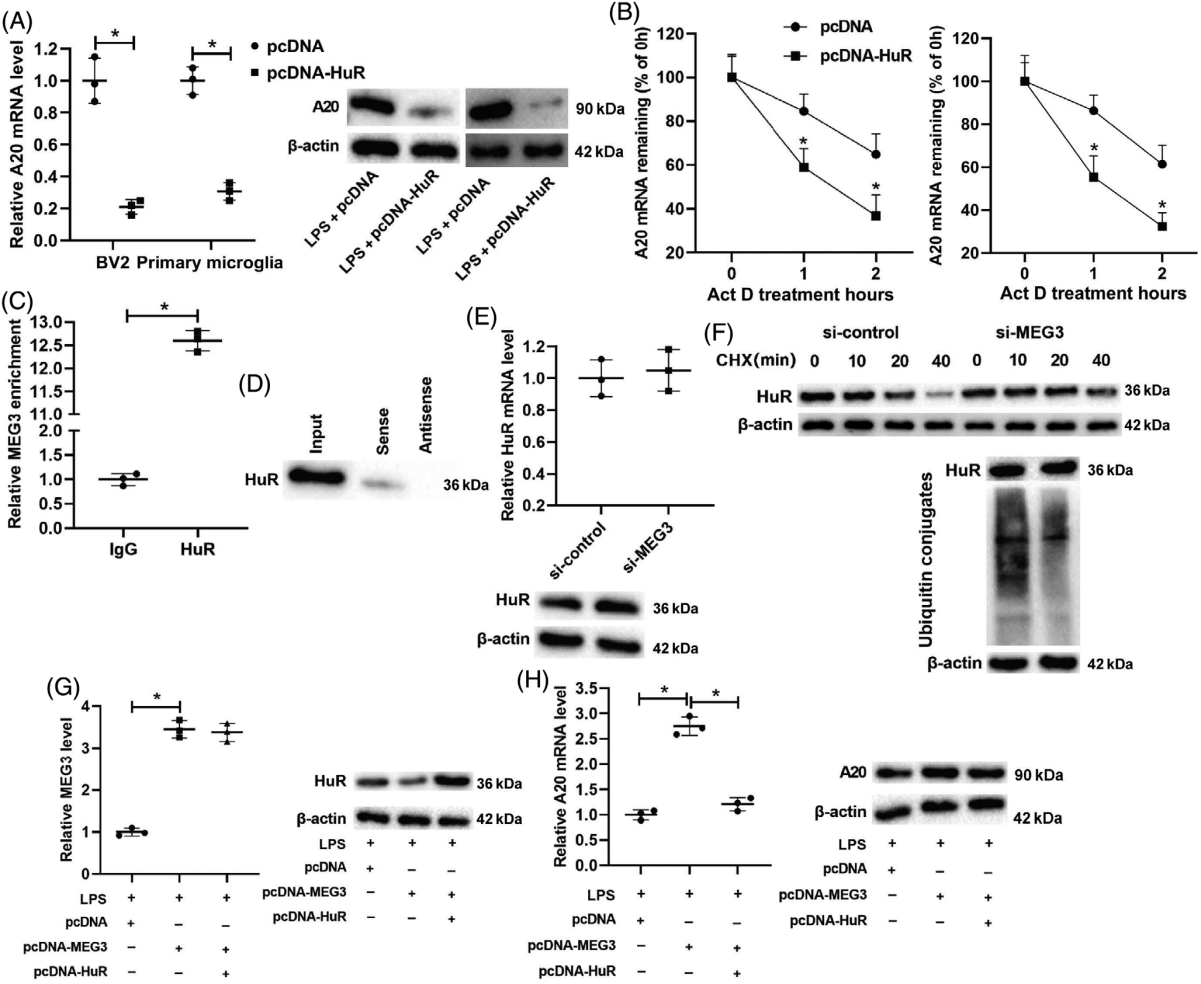
HuR regulates the degradation of A20 mRNA and verification of the interaction between lncRNA MEG3 and HuR protein. (A) pcDNA‐HuR was transfected into BV2 cells and primary microglia for 48 h and then treated the cells with LPS for 24 h. qRT‐PCR and western blot were applied to test the mRNA and protein levels of A20. (B) pcDNA‐HuR was transfected into BV2 cells and primary microglia and then treated cells with an RNA synthesis inhibitor Actinomycin D for 0, 1, and 2 h. qRT‐PCR was performed to measure the mRNA level of A20. (C,D) RNA immunoprecipitation (RIP) and RNA pull‐down assays in BV2 cells were conducted to confirm the interaction between lncRNA MEG3 and HuR protein. (E) si‐MEG3 was transfected into BV2 cells. qRT‐PCR and western blot were applied to test the mRNA and protein levels of HuR. (F) si‐MEG3 was transfected into BV2 cells and then treated cells with a protein synthesis inhibitor cycloheximide (CHX) for 0, 10, 20, and 40 min. CHX‐chase and ubiquitination assays were applied to analyze the ubiquitination degradation of the protein. (G) pcDNA‐MEG3 and/or pcDNA‐HuR was transfected into BV2 cells, and the cells were treated with LPS for 24 h. qRT‐PCR was applied to measure the expression of lncRNA MEG3, and western blot was performed to test the protein level of HuR. (H) qRT‐PCR and western blot were conducted to test the mRNA and protein levels of A20. *P < 0.05 vs. LPS + pcDNA, pcDNA, IgG, or LPS + pcDNA‐MEG3. Data are represented as the mean ± SD of three independent assays

To further probe into the potential mechanisms, pcDNA‐HuR was transfected into BV2 cells and primary microglia for 48 h, and then the cells were treated with LPS for 24 h. The qRT‐PCR and Western blot results showed that the overexpression of HuR lowered the mRNA and protein levels of A20 (Figure 5A). Actinomycin D is an RNA synthesis inhibitor [35]. pcDNA‐HuR was transfected into BV2 cells and primary microglia, and then the cells were treated with actinomycin D for 0, 1, and 2 h. As shown in Figure 5B, the overexpression of HuR boosted the degradation of A20 mRNA.

Combining the finding shown in Figure 5E that lncRNA MEG3 positively regulated the mRNA and protein levels of A20 in BV2 cells with the finding from the bioinformatics software analysis that lncRNA MEG3 might bind to HuR protein, we speculated that lncRNA MEG3 might influence the mRNA level of A20 through its interaction with HuR protein, and this speculation was further confirmed by the results of the RIP and RNA pull‐down assays in BV2 cells (Figure 5C,D). Moreover, si‐MEG3 was transfected into BV2 cells. As shown in Figure 5E, the interference with lncRNA MEG3 raised the protein level of HuR but had no significant influence on its mRNA level. CHX is a widely used inhibitor of protein synthesis [36]. si‐MEG3 was transfected into BV2 cells, and then the cells were treated with CHX for 0, 10, 20, and 40 min. As shown in Figure 5F, the interference with lncRNA MEG3 restrained the ubiquitination degradation of HuR protein.

Furthermore, pcDNA‐MEG3 and/or pcDNA‐HuR was transfected into BV2 cells, and the cells were treated with LPS for 24 h. The qRT‐PCR analysis expounded that the overexpression of lncRNA MEG3 raised the lncRNA MEG3 expression while the overexpression of HuR did not affect the lncRNA MEG3 expression. Furthermore, the Western blot assay indicated that the overexpression of lncRNA MEG3 lowered the HuR protein level while the overexpression of HuR reversed this trend (Figure 5G). The overexpression of lncRNA MEG3 also raised the mRNA and protein levels of A20 while the overexpression of HuR lowered the A20 mRNA level but did not affect the A20 protein level (Figure 5H). In general, these results demonstrated that HuR promoted the degradation of A20 mRNA, and lncRNA MEG3 boosted the ubiquitination degradation of HuR protein by binding to it.

### 3.5 lncRNA MEG3 restrains the M1 polarization of microglia through the HuR/A20/NF‐κB axis

**FIGURE 6:**
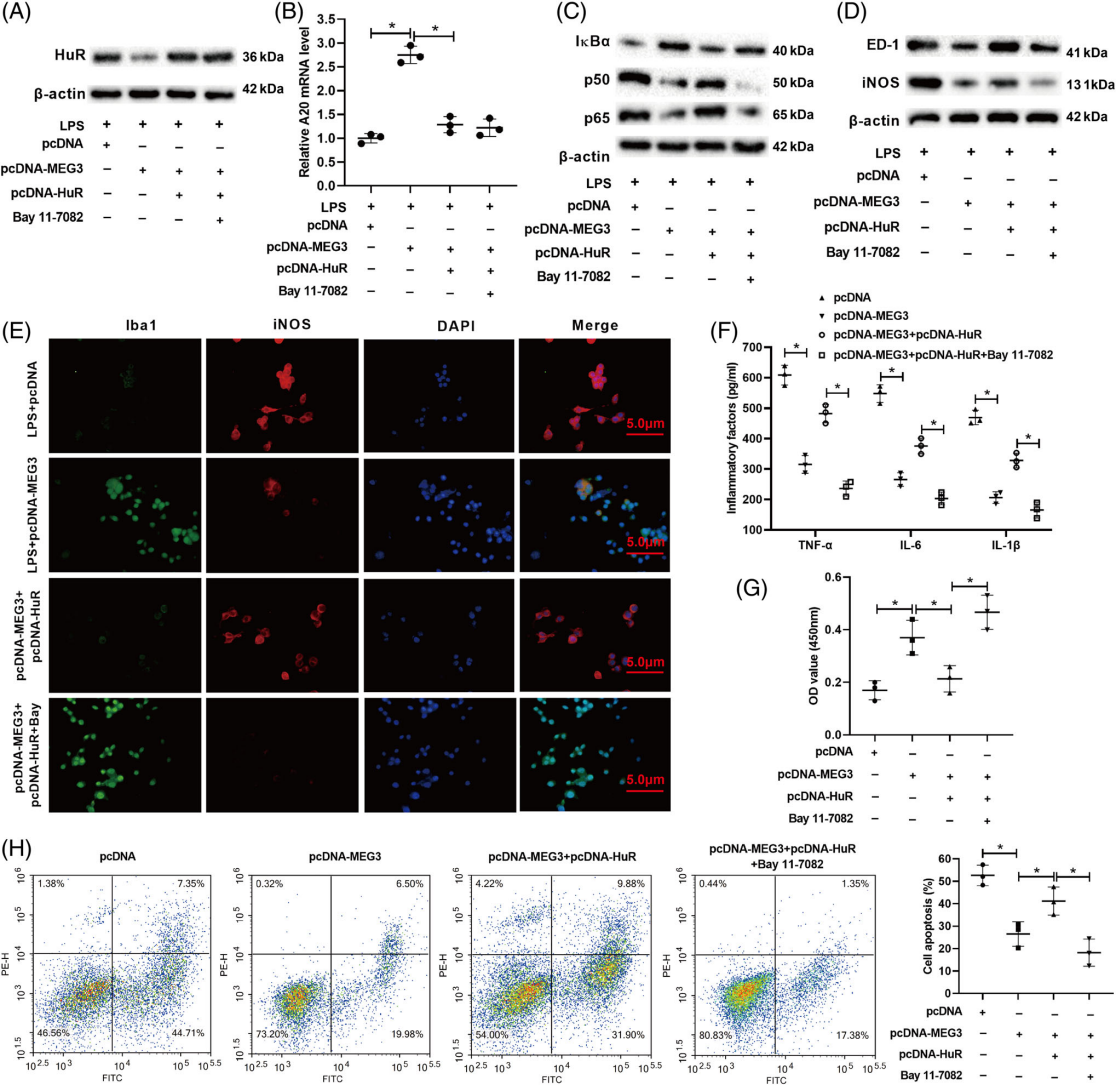
lncRNA MEG3 affects the M1 polarization of microglia through the HuR/A20/NF‐κB axis. (A) pcDNA‐MEG3 and/or pcDNA‐HUR was transfected into BV2 cells and the cells were treated with an NF‐κB pathway inhibitor BAY 11‐7082, and the cells were further treated with LPS for 24 h. Western bolt was applied to detect the protein level of HuR. (B) qRT‐PCR was applied to measure the mRNA level of A20. (C,D) Western bolt was applied to test the protein levels of IκBα, p50, p65, ED‐1, and iNOS. (E) Immunofluorescence assay was applied to analyze the expressions of Iba‐1 and iNOS (scale bar: 5 μm). (F) ELISA was applied to detect the concentrations of TNF‐α, IL‐6, and IL‐1β. (G) BV‐2 cells were co‐incubated with human nerve cells SH‐SY5Y for 12 h. CCK‐8 was applied to assess the proliferation of SH‐SY5Y cells. (H) Flow cytometry assay was applied to analyze the apoptosis of SH‐SY5Y cells. *P < 0.05 vs. LPS + pcDNA, LPS + pcDNA‐MEG3, pcDNA, or pcDNA‐MEG3 + pcDNA‐HuR. Data are represented as the mean ± SD of three independent assays

To verify if lncRNA MEG3 regulated the M1 polarization of microglia via the HuR/A20/NF‐κB axis, pcDNA‐MEG3 and/or pcDNA‐HuR was transfected into BV2 cells, and the cells were treated with the BAY 11–7082 NF‐κB pathway inhibitor and were further treated with LPS for 24 h. As shown in Figure 6A, the overexpression of HuR reversed the lowering of the HuR protein level caused by the overexpression of lncRNA MEG3 while the treatment with Bay 11‐7082 did not affect the HuR protein level. The overexpression of HuR reversed the rise of the A20 mRNA level caused by the overexpression of lncRNA MEG3 while the treatment with Bay 11‐7082 did not affect the A20 mRNA level (Figure 6B). Furthermore, the overexpression of HuR reversed the rise of the IκBα protein level and the lowering of the p50 and p65 protein levels caused by the overexpression of lncRNA MEG3, but these trends were reversed by the treatment with Bay 11‐7082 (Figure 6C). Besides, the overexpression of HuR reversed the lowering of the ED‐1 and iNOS protein levels caused by the overexpression of lncRNA MEG3, but these trends were reversed by the treatment with Bay 11‐7082 (Figure 6D). As shown in Figure 6E, the overexpression of HuR reversed the increase in Iba‐1 expression and the decrease in iNOS expression caused by the overexpression of lncRNA MEG3, but these trends were reversed by the treatment with Bay 11‐7082. Furthermore, the overexpression of HuR reversed the decrease in the TNF‐α, IL‐6, and IL‐1β concentrations caused by the overexpression of lncRNA MEG3, but these trends were reversed by the treatment with Bay 11‐7082 (Figure 6F).

BV‐2 cells were co‐incubated with the SH‐SY5Y human nerve cells for 12 h. As shown in Figure 6G, the overexpression of HuR reversed the boosting of the SH‐SY5Y cell proliferation caused by the overexpression of lncRNA MEG3, but this trend was reversed by the treatment with Bay 11‐7082. On the contrary, the overexpression of HuR reversed the restraint in the SH‐SY5Y cell apoptosis caused by the overexpression of lncRNA MEG3, but this trend was reversed by the treatment with Bay 11‐7082 (Figure 6H). To sum up, lncRNA MEG3 restrained the M1 polarization of microglia through the HuR/A20/NF‐κB axis.

### 3.6 Effects of the overexpression of lncRNA MEG3 on motor function recovery and neuroinflammation relief in mice with SCI

**FIGURE 7:**
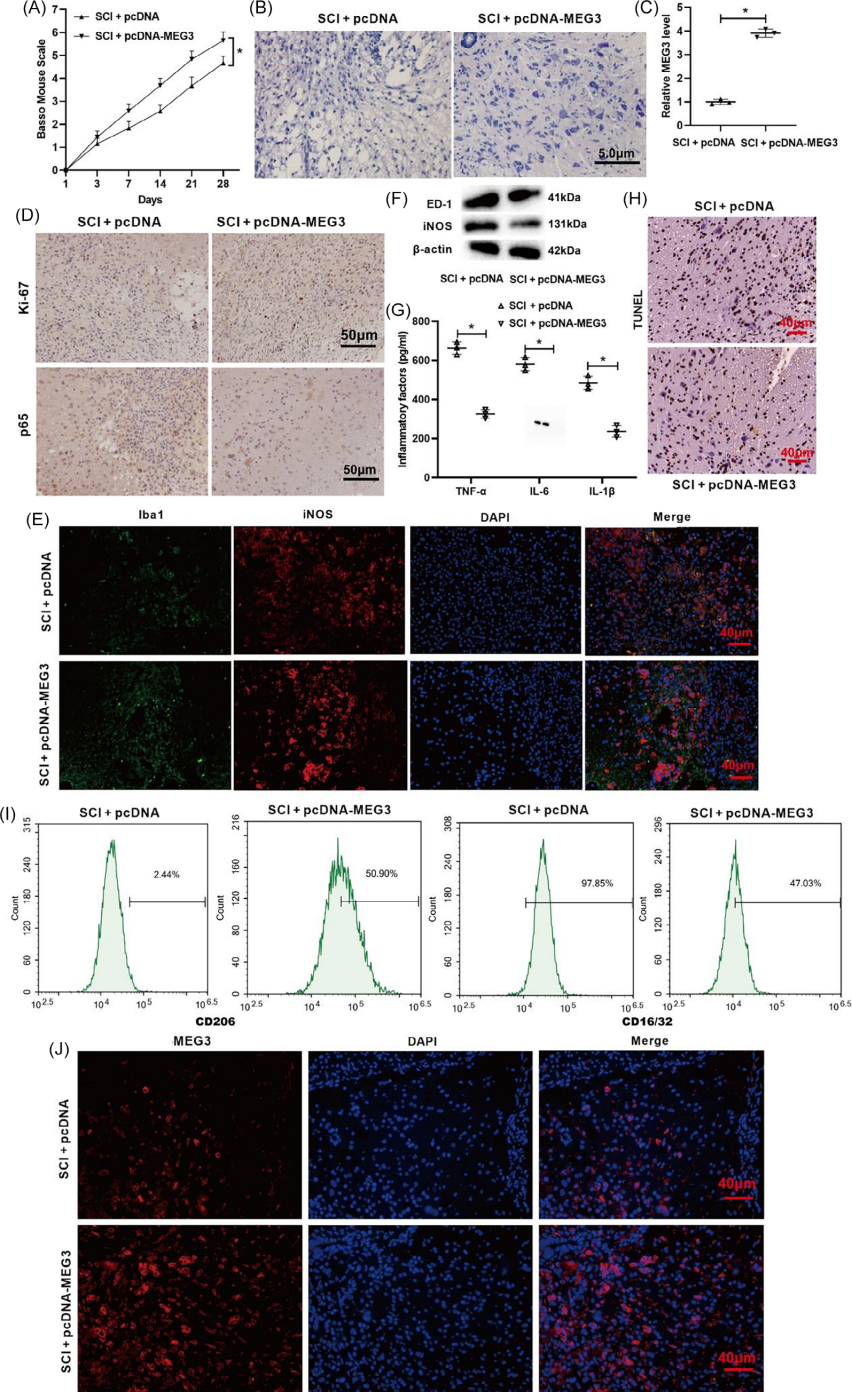
Influence of lncRNA MEG3 on motor function recovery and neuroinflammation relief in SCI mice. (A) The mouse model of SCI was constructed and pcDNA‐MEG3 was injected intrathecally into mice. BMS score was performed to analyze the motor function of mice. (B) Nissl staining was conducted to assess the pathological characteristics of spinal cord tissues in mice (scale bar: 5 μm). (C) qRT‐PCR was conducted to test the expression of lncRNA MEG3. (D) The immunohistochemical assay was applied to detect the expressions of Ki‐67 (a marker for proliferation) and p65 (scale bar: 50 μm). (E) Immunofluorescence was applied to analyze the expressions of Iba1 and iNOS (scale bar: 40 μm). (F) Western blot was performed to test the protein levels of ED‐1 and iNOS. (G) ELISA was applied to measure the concentrations of TNF‐α, IL‐6, and IL‐1β. (H) TUNEL assay was conducted to assess the cell apoptosis (scale bar: 40 μm). *P < 0.05 vs. SCI + pcDNA. SCI, spinal cord injury. (I) Primary mouse microglia were isolated from the normal mouse and cultured. CD206 (M2) and CD16/32 protein expressions were measured by flow cytometry assay. (J) RNA fluorescence in situ hybridization (RNA FISH) assay was used to detect MEG3 expression in mouse spinal cord tissues. Data are represented as the mean ± SD of three independent assays [Correction added on 1 August 2022, after first online publication: Figure 7 has been replaced.]

Next, we verified the role of lncRNA MEG3 in motor function recovery and neuroinflammation alleviation in mice with SCI. As shown in Figure 7A, the overexpression of lncRNA MEG3 raised the BMS score, hinting that the overexpression of lncRNA MEG3 boosted the mice's motor function recovery. Similar to this conclusion, the overexpression of lncRNA MEG3 reversed the reduction of Nissl bodies and some neural shrinkages induced by SCI (Figure 7B). Moreover, the expression of lncRNA MEG3 in the spinal cord tissues indeed increased after the overexpression of lncRNA MEG3 (Figure 7C).

Ki‐67 is a widely applied marker for proliferation [37]. The immunohistochemical results showed that the overexpression of lncRNA MEG3 increased the Ki‐67 expression and lessened the p65 expression, implying that the overexpression of lncRNA MEG3 boosted the cell proliferation and restrained the activation of the NF‐κB signaling pathway (Figure 7D). The immunofluorescence results showed that the overexpression of lncRNA MEG3 increased the Iba1 expression and lessened the iNOS expression (Figure 7E). Furthermore, the overexpression of lncRNA MEG3 lowered the protein levels of ED‐1 and iNOS, suggesting that the overexpression of lncRNA MEG3 repressed the activation of microglia (Figure 7F). As shown in Figure 7G, the overexpression of lncRNA MEG3 reduced the TNF‐α, IL‐6, and IL‐1β concentrations. In addition, the overexpression of lncRNA MEG3 restrained cell apoptosis (Figure 7H). The membrane protein CD16/32 (M1) was decreased and CD206 (M2) was increased after lncRNA MEG3 overexpression (Figure 7I). RNA FISH of mouse spinal cord tissue showed that MEG3 was mainly expressed in the cytoplasm of nerve cells and microglia (Figure 7J). In general, these results indicate that the overexpression of lncRNA MEG3 boosted motor function recovery and neuroinflammation relief in the mice with SCI in this study.

## 4 DISCUSSION

The current study aimed to reveal the function and underlying mechanism of lncRNA MEG3 in the M1 polarization of microglia and neuroinflammation. In this study, we discovered that the lncRNA MEG3 expression was lessened in the ASCI mouse spinal cord tissues and LPS‐treated microglia. Based on this finding, we further demonstrated that the overexpression of lncRNA MEG3 restrained the M1 polarization of the microglia and the neuroinflammation caused by LPS. The investigation of the underlying mechanisms expounded that lncRNA MEG3 regulated the M1 polarization of the microglia through the HuR/A20/NF‐κB axis and that the overexpression of lncRNA MEG3 boosted the motor function recovery and neuroinflammation relief in the mice with SCI (summarized in Graphical abstract).

As is widely known, the imbalance of the M1 polarization of microglia and abnormal neuroinflammation play pivotal functions in ASCI [38, 39]. Thus, this study was conducted to probe into the underlying molecular mechanisms and associated signaling pathways in the restraint of the M1 polarization of microglia and neuroinflammation induced by LPS. Recently, an increasing number of studies have shown that lncRNAs are bound up with the process of ASCI by regulating the inflammation‐related signaling pathways and inflammatory response [40, 41]. lncRNA MEG3, the first lncRNA confirmed to have a tumor suppressor function, has been found to have an important regulatory function in neurological diseases such as glioma and ischemic stroke [42, 43]. Crucially, the expression profile of lncRNAs in the mouse SCI dataset GSE45376 revealed that the lncRNA MEG3 expression was lessened after SCI occurrence. On the basis of this finding and of the relevant research results on the function of lncRNA MEG3, we confirmed that the overexpression of lncRNA MEG3 restrained the M1 polarization of microglia and neuroinflammation.

HuR, also named Elavl1, is an RNA‐binding protein that can bind to the AU‐rich elements (AREs) in the 3′ prime untranslated region (3′‐UTR) of its target mRNAs and can raise mRNA stability and translation by competing for ARE occupancy against mRNA‐destabilizing modulators [44, 45]. Previous studies have confirmed that the enhanced HuR expression in astrocytes exacerbates neuronal injury and SCI [46] and that the translocation of HuR in astrocytes promotes inflammatory responses in SCI [47]. Similar to these findings, our studies also corroborated that lncRNA MEG3 bound to HuR and negatively regulated the expression of HuR, indicating that lncRNA MEG3 could regulate the M1 polarization of microglia and neuroinflammation through HuR.

A20 is a zinc finger protein that is a potent regulator of ubiquitin (Ub)‐dependent signals [48]. Studies have indicated that HuR binds to the 3′‐UTR of A20 and recruits RNA‐induced silencing complex to boost A20 mRNA degradation [49]. As expected, our study also confirmed that HuR boosted the degradation of A20 mRNA. As has been reported, A20 represses the activation and transfer of the nuclear transcription factor NF‐κB into the nucleus [50], and crucially, NF‐κB plays key roles in the process of microglia M1 polarization to cytokine release, and the activation of NF‐κB boosts the M1 polarization of microglia, leading to a neuroinflammatory response [51]. Similarly, we confirmed that lncRNA MEG3 regulated the M1 polarization of microglia through the HuR/A20/NF‐κB axis.

Notably, there are still some limitations in this study. It is difficult to collect clinical samples, so the sample size is not sufficient. We will expand clinical data in future studies, so that the conclusions of this article will be more evidence‐based. In general, the data that we obtained in this study showed that lncRNA MEG3 functioned in the M1 polarization of microglia and neuroinflammation, and provided a novel regulation axis in ASCI: lncRNA MEG3/HuR/A20/NF‐κB. Our study might provide novel molecules and insights for the restraint of the M1 polarization of microglia and of neuroinflammation to relieve ASCI.

## CONFLICT OF INTEREST

The authors have no conflicts of interest to disclose.

## Supporting information


**Figure S1** Peripheral blood samples were collected from patients with traumatic spinal cord injury (SCI, *n* = 36) and healthy control (*n* = 36) and the serum samples were centrifuged at 3000 rpm for 10 min. (A) The expression of lncRNA MEG3 was tested by qRT‐PCR. (B) The concentrations of TNF‐α, IL‐6, and IL‐1β were detected by ELISA assay. (C) Correlation analysis was conducted between the expression of lncRNA MEG3 and the levels of inflammatory factors TNF‐α, IL‐6, and IL‐1β. *P < 0.05, *** *P* < 0.001 versus Healthy. Data are represented as the mean ± SD of three independent assays.

## Data Availability

Data sharing is not applicable to this article as no new data were created or analyzed in this study.
