# Supplementary figures and images for: lncRNA MEG3 restrained the M1 polarization of microglia in acute spinal cord injury through the HuR/A20/NF‐κB axis
Source: Brain Pathol. 2022 Mar 25;32(5):e13070. doi: 10.1111/bpa.13070 (PMC9425005; doi:10.1111/bpa.13070)

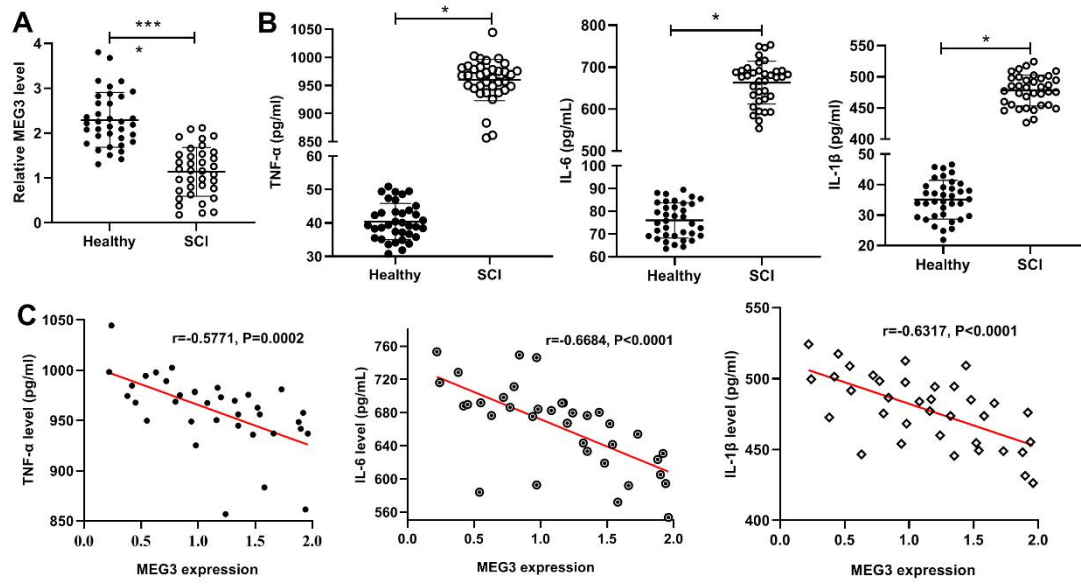

Supplement: Supplementary file 1 — Figure S1 Peripheral blood samples were collected from patients with traumatic spinal cord injury (SCI, n = 36) and healthy control (n = 36) and the serum samples were centrifuged at 3000 rpm for 10 min. (A) The expression of lncRNA MEG3 was tested by qRT‐PCR. (B) The concentrations of TNF‐α, IL‐6, and IL‐1β were detected by ELISA assay. (C) Correlation analysis was conducted between the expression of lncRNA MEG3 and the levels of inflammatory factors TNF‐α, IL‐6, and IL‐1β. *P < 0.05, *** P < 0.001 versus Healthy. Data are represented as the mean ± SD of three independent assays. [file BPA-32-e13070-s001.pdf]
